# Fostering Digital Well-Being Through (e-)Service-Learning: Engaging Students in Responsible and Inclusive Digital Practices

**DOI:** 10.3390/bs15091158

**Published:** 2025-08-25

**Authors:** Irene Culcasi, Rosario Cerrillo, Maria Cinque

**Affiliations:** 1 Department of Human Sciences, LUMSA University of Rome, 00193 Roma, Italy; i.culcasi@lumsa.it (I.C.); m.cinque@lumsa.it (M.C.); 2 Departamento de Pedagogía, Autonomous University of Madrid, 28049 Madrid, Spain

**Keywords:** digital well-being, service-learning, e-service-learning, youth participation, experiential education, educational technology, quality standards, case studies, review

## Abstract

(1) Background: In today’s digital society, challenges like cyberbullying, harmful social media use, and unhealthy digital habits demand innovative and inclusive educational responses. This study investigates the potential of service-learning (SL) and electronic service-learning (e-SL) as experiential approaches to enhance digital well-being among youth. By actively engaging students, educators, and community stakeholders in co-designed projects, SL/e-SL promotes critical awareness, digital citizenship, and prosocial values while addressing digital risks. (2) Methods: This review offers a literature-based analysis of existing programs and good practices that apply experiential education to encourage responsible digital engagement. It explores SL and e-SL experiences across various educational settings. (3) Results: The findings show that SL and e-SL can be effective educational tools, creating meaningful opportunities for youth to participate in tackling digital issues and building inclusive spaces where students, faculty, and communities collaborate to foster digital literacy and well-being. The analysis also led to the development of quality standards for SL and e-SL practices that promote digital well-being. (4) Conclusions: This study highlights key implications for teaching, underscoring the value of integrative pedagogies that connect experiential learning to digital challenges, promoting a more inclusive and responsible digital culture.

## 1. Introduction

In just a few decades, service-learning (SL) has evolved from a relatively unknown pedagogical approach into one of the most influential educational strategies (Paz-Lourido & De-Benito, 2021). As Eyler and Giles (2007) state, SL emerged to combine the potential benefits of experiential learning and community service. While SL itself is not new, a recent innovation is the balanced planning of SL projects in a close interaction with technologies, an acceleration due to the COVID-19 pandemic. According to Sparkman et al. (2020), technology has come to the SL to stay; it is therefore necessary to focus pedagogical reflections toward the search on the current state of this relationship, looking for quality standards for what is now known as electronic service-learning (e-SL).

Definitions of e-SL in the literature emphasize different various aspects of the educational approach, emphasizing specific elements:

- e-SL occurs when the instructional component and the service component in integrative pedagogy engage learners through technology in civic inquiry, service, reflection, and action, and are both conducted online (Marcus et al., 2019);
- e-SL, as a special kind of SL, additionally recognizes the emerging role of technology in shaping students’ participation in the community and provides a quality experience while meeting the needs of multiple participants from multiple grounds, giving them the ability to make connections across disciplines (Modic Stanke et al., 2021);
- e-SL combines digital technology with social services to further improve the quality of civic engagement and bridge digital gaps in the local community (Semenski et al., 2017).

Whether SL in an online environment is feasible is a legitimate question. Indeed, as Cook-Benjamin (2015) states, adapting SL from the in-person to the online environment is not easy because SL is an immersive, challenging and highly personally involved activity. Therefore, in order to determine whether or not SL is viable in an asynchronous or distal learning environment, we need to understand what the obstacles and challenges are. As Guthrie and McCracken (2010) state, creating a virtual teaching and learning environment is a challenge in itself. It requires capturing and nurturing both teaching moments that occur during planned web-based activities and spontaneous service-based learning experiences.

The literature in the field identifies three main areas of challenge in e-SL related to digital well-being: participation, relational dynamics, and technology and impact.

- Participation: Students often struggle to feel part of the community they are serving, especially in the absence of direct human contact (Cook-Benjamin, 2015; Schwehm et al., 2017). Peer motivation is harder to activate in a virtual environment, and group work is often unbalanced, with frequent instances of “free-riding”—where some students benefit from others’ efforts without contributing themselves (Harris, 2017). The lack of familiarity and direct interaction among students, faculty, and community partners further limits the development of leadership and effective collaboration (Cook-Benjamin, 2015).
- Relational Dynamics: The virtual setting alters important interpersonal dynamics that are essential in SL. In large online classes, students may be reluctant to share personal reflections (Guthrie & McCracken, 2014), while the absence of face-to-face conversation makes it more difficult to express one’s perspective (Shah et al., 2018). Moreover, participation is often passive: students may log in but not truly engage, creating an imbalance in the learning process and centralizing the instructor’s role (L. Waldner et al., 2010). Building trust and rapport becomes more challenging, particularly for those unfamiliar with SL (Cook-Benjamin, 2015; Seru, 2021).
- Technology and Impact: Studies report recurring technological issues such as unstable internet connections, outdated equipment, or inadequate platform management (Chen et al., 2011; Lin & Shek, 2021). The more central technology becomes, the more the human connection may be compromised (Harris, 2017). Personalized monitoring of student projects is difficult to achieve (García-Gutiérrez et al., 2021), and the use of indirect SL approaches risks reducing the real impact on communities (Lin & Shek, 2021).

In sum, students’ digital well-being and the quality of educational relationships are both challenged in e-SL environments, calling for thoughtful pedagogical strategies, stronger relational support, and attention to technological accessibility. Moreover, if we consider technology as a tool that students use to respond to community needs and collaborate with community members—at varying levels of complexity—or if we view technology as the central topic of a project addressing risks and well-being in the digital sphere, a significant gap in the literature becomes evident. Most studies treat technology merely as a functional channel that neither requires advanced digital literacy nor calls for intentional integration from a pedagogical standpoint, as if technology were simply an alternative means of engaging with the community when in-person interaction is not possible (Culcasi et al., 2022a). As a result, while concepts such as digital well-being and digital citizenship may be present at a theoretical level (Escofet, 2025), they are rarely embedded into actual project design and are not supported by spaces for intentional reflection.

This study explores the potential of e-SL as an experiential method to foster digital well-being among young people. By actively engaging students, educators, and community stakeholders in co-designed initiatives, e-SL enhances critical awareness, strengthens digital citizenship and nurtures prosocial values while addressing the risks associated with digital environments (Brozmanová-Gregorová et al., 2025). This co-design process is not merely a methodological choice but a pedagogical strategy that fosters ethical collaboration and shared responsibility among all actors involved (Mould, 2014). When thoughtfully structured, co-creation in e-SL becomes a dynamic, multidimensional practice that supports the development of inclusive, digitally competent communities. It enables students to move beyond the passive consumption of digital content, cultivating instead the capacity to critically engage, collaborate and co-produce knowledge and solutions with real-world impact (Bidar, 2018). According to De Koning et al. (2016), several theoretical frameworks on co-creation emphasize the importance of actor roles, participation intensity, and digital platform capabilities. These frameworks offer useful insights for designing e-SL experiences that promote digital well-being in a responsible and contextualized way.

To frame the study’s scope and contribution, the investigation begins by outlining the conceptual framework of digital well-being, with specific reference to the educational and civic goals of SL and e-SL. It examines the evolution of SL into its digital counterpart, identifying e-SL as a pedagogical response to both the opportunities and challenges posed by digital transformation in higher education.

Specifically, the second section explores three core dimensions: SL as a method of civic engagement and experiential learning; e-SL as its digital adaptation, including specific typologies and practices; and digital well-being as a multidimensional construct encompassing emotional, cognitive, and relational aspects of online engagement.

These dimensions are then interwoven to emphasize how e-SL can be intentionally designed to promote students’ digital well-being through reflective practices, inclusive digital learning environments, and the ethical use of technology. Special attention is given to the digital competencies required by both students and educators to ensure meaningful and equitable participation.

The central part of the article presents a case study analysis of three academic initiatives that integrate digital well-being into SL and e-SL frameworks. Drawn from diverse institutional contexts, these cases are examined to extract quality standards, pedagogical strategies, and context-specific challenges.

In the final sections, the article reflects on emerging trends, ethical considerations, and the need for teacher training programs that promote both digital literacy and socio-emotional skills. The contribution concludes with recommendations for systematically embedding digital well-being within SL practices and suggests future research directions.

## 2. Theoretical Framework

### 2.1. Service-Learning

SL is more than a methodology or a teaching strategy; it is conceived as a way of understanding education, a lens through which to critically observe reality, and a pedagogical approach that permeates the entire educational process. Rooted in social needs and aligned with curricular learning objectives, SL enables students to serve their communities by applying acquired knowledge and engaging in critical reflection on their experiences (Batlle, 2020; Puig, 2018). Consequently, the teaching−learning process promoted through SL extends beyond the classroom, requiring collaboration with community and social actors. SL, thus, constitutes a coherent and transformative educational project that opens up opportunities for students to address real-world problems and contribute to social improvement.

Among the most relevant proposals for educational renewal, the UNESCO Report *Reimagining our futures together: A new social contract for education* (United Nations Educational, Scientific and Cultural Organization [UNESCO], 2021) calls for curricula to emphasize ecological, intercultural, and interdisciplinary learning, supporting students in both acquiring knowledge and developing the ability to critically analyze and apply it. Achieving this vision demands that higher education institutions be meaningfully engaged in their local communities.

The UNESCO Report states that forging peaceful, just, and sustainable futures requires a fundamental transformation of education. Looking ahead to 2050, it argues that any new social contract for education must be based on two core principles: guaranteeing the right to quality education throughout life and reinforcing education as a public and common good. These principles are essential to ensuring that education empowers future generations to reimagine and shape their futures.

SL is a powerful tool to support this transformation. It promotes social change and fosters sustainable development and social justice (Aramburuzabala et al., 2024), engaging students, educators, and community members in meaningful, active, and participatory learning processes.

By linking academic knowledge with social action—especially within digital environments—SL encourages the development of responsible digital competencies, supports youth-led initiatives and promotes critical, global forms of civic engagement that go beyond passive media consumption (Escofet, 2025; Cajavilca et al., 2025). In this way, SL contributes to the emergence of a new generation of civic-minded young people, capable of navigating complex digital ecosystems while advocating for equity, justice, and social transformation.

Among the numerous benefits that SL offers to the learning process, one of the most widely acknowledged by the scientific community is its capacity to support the development of curricular competencies (López-de-Arana et al., 2019). Research has shown that complex concepts and ideas are more effectively retained when learning is connected to lived experience, thereby enhancing the transfer of knowledge and skills to real-world contexts. By encouraging reflection on authentic experiences, SL contributes to deeper understanding and improved recall of complex content. This effect is amplified when students feel a sense of responsibility for their own learning, which has been linked to enhanced academic performance. In addition to discipline-specific learning, SL has been shown to promote the development of key transversal competencies such as autonomy, collaboration, entrepreneurship, critical thinking, social competence, civic engagement, and a sense of social responsibility (Aramburuzabala et al., 2019; Culcasi & Paz Fontana Venegas, 2023). It also nurtures greater awareness of social justice by encouraging students to critically examine societal structures and to value transformative action over charitable intervention. In this regard, SL calls for a posture of humility from students, who are invited to approach community engagement not as “experts” providing help, but as learners engaging in mutual exchange. Recognizing that one often receives as much—if not more—than one gives is central to the SL experience (Cerrillo & McIlrath, 2022). This reciprocity fosters a sense of accomplishment and satisfaction among students involved in SL projects, particularly when they perceive tangible outcomes from their efforts. Such experiences often lead students to become more aware of their potential as active agents of social change.

### 2.2. e-Service-Learning: Toward a Definition

e-service-learning is a technology-enhanced educational approach that combines academic learning with social commitment. This implies the following: serving the community to address real-world challenges by leveraging academic disciplines and personal, social, methodological and digital skills; reflecting on the service experience; and then learning through the process.

Drawing on the same foundations as SL, e-SL goes beyond volunteering and is closely tied to curricular learning, as it integrates academic content with meaningful community engagement through structured, reflective, and outcome-oriented pedagogical processes. It is not merely a classroom-based study activity, but rather involves applying acquired knowledge to real-world challenges instead of artificial tests. It represents a true pedagogical paradigm shift, fostering a rethinking of the curriculum through the lens of real-world impact and improvement. The digital component further enhances this approach, offering various levels of implementation and outcomes. In particular, the technology dimension of e-SL acts on two levels: implementation and outcome.

At the implementation level—considering technology as a learning environment—e-SL enables participation even when physical access is not possible. Various hybrid forms exist, such as when service is in-person and instruction is online (e-SL hybrid type 1), or vice versa (e-SL hybrid type 2), and a mix of partly in-person and online activities for both service and learning (e-SL hybrid type 3), leading up to 100% online SL (extreme e-SL) (L. S. Waldner et al., 2012).

At the outcome level—considering technology as a tool that can be integrated with varying complexity within e-SL projects—different forms of technology use have been identified (Culcasi et al., 2022b):

- *Instrumental Channel*: Technology is a basic channel of remote communication to reach the community, but it is not directly connected to the learning or service goals of the project (e.g., using a messaging platform to coordinate the logistics of distributing goods to vulnerable families in remote areas);
- *Integrated Channel*: technology as a channel intentionally linked to the project goals (e.g., designing and disseminating educational content through a thematic blog to promote digital health among adolescents);
- *Instrumental Objective*: Technology is part of the product of the project, including existing digital tools and resources (e.g., creating a YouTube channel with multilingual video tutorials to support the digital literacy of migrants and refugees);
- *Integrated Objective*: Technology is the ultimate goal of the project, requiring high-level expertise to develop technologically innovative solutions (e.g., developing a mobile application that enables citizens and organizations to map, in real time, situations of environmental or social distress in marginalized urban areas). An interesting example of this most complex technological interaction is IARA, an AI-based system designed by Argentinian students to detect tuberculosis, a critical health issue affecting vulnerable communities (Gil Moreira, 2023). Through this initiative, students collaborated with medical professionals and leveraged artificial intelligence to create a diagnostic app that enhances early detection and treatment accessibility. The project exemplifies how technology and AI, when applied intentionally within an e-SL framework, redefine learning as an interactive process of discovery and social transformation. By listening to community needs and taking concrete action, these students demonstrated the power of technology-mediated human connection, embodying the principles of e-SL through direct engagement with real societal issues. This case highlights how e-SL can integrate technologies not only as a tool but as a means to drive civic engagement and social change.

It is important to consider that e-SL represents a model which recognizes the complex and dynamic interconnection between digital technologies, teaching, educational purposes, ethical values, and the sociocultural context in which education takes place. This aligns with Escofet’s (2025) concept of *entangled pedagogy* (Fawns, 2022) which strongly critiques the traditional view that pedagogy and technology are independent elements or that one should be subordinated to the other. Instead, it proposes a holistic and integrated vision of the two elements, which constantly interact with the factors of the educational context. Technology is integrated into educational processes in a meaningful and reflective way, promoting a dimension of digital citizenship that is closely linked to digital well-being, understood not as a passive reaction to technologies, but as an active construction through them in terms of the following: competences, critical reflection, individual and collective participation, digital rights and norms, as well as civic responsibility. Definitely, the SL approach, enhanced by digital technologies, offers a pathway to integrate digital citizenship as part of the educational process, enabling the actors involved not only to develop digital skills but also to actively experience digital citizenship, contributing to their own well-being and that of the communities.

### 2.3. Defining Digital Well-Being and Its Key Dimensions

Recent literature reflects growing interest in the concept of digital well-being, particularly as digital technologies increasingly mediate daily life, health, education, and social interaction. Although there is no widely accepted definition, scholars generally agree that digital well-being refers to an individual’s ability to maintain a balanced, healthy, and mindful relationship with digital tools—one that supports emotional, physical, and social flourishing (Al-Mansoori et al., 2023; Büchi, 2024; Smits et al., 2022). From a theoretical standpoint, Büchi (2024) conceptualizes digital well-being as a subdimension of subjective well-being in the context of ubiquitous digital media, emphasizing the need to balance the benefits and harms of digital practices. Similarly, Dewitz (2022) highlights the role of self-regulation, information literacy, and mindful media use in maintaining digital well-being, identifying three core components: learning about well-being, quantifying it through self-tracking, and achieving it via intentional digital practices.

Digital well-being is broadly defined as a state of mental, physical, and social health shaped by one’s interaction with digital environments. It includes not only the prevention of harm (e.g., addiction, cyberbullying), but also the promotion of flourishing through technology use (Al-Mansoori et al., 2023; Smits et al., 2022). Empirical research supports these frameworks by showing that excessive or unmoderated digital engagement can negatively impact emotional and physical health. For example, Almourad et al. (2021) found that users of digital well-being apps report benefits such as reduced anxiety and improved focus, though the success of such tools depends on usability and user trust. Bhattacharya et al. (2023) extended this by emphasizing the psychological risks of digital overexposure—such as fear of missing out (FOMO) and nomophobia—and proposed leveraging digital footprints to support well-being interventions. This refers to the idea of using the data generated by individuals’ online activities (the so called “digital footprints”) to design and implement interventions aimed at improving their well-being. One key dimension is mental and emotional well-being, which includes the psychological effects of digital use, such as stress, anxiety, and self-esteem. Overexposure to digital environments can lead to outcomes like depression and social withdrawal (Bhattacharya et al., 2023; Thomas et al., 2022).

Physical well-being is also impacted, particularly in relation to sleep quality, eye strain, posture, and sedentary behavior. Pediatric and educational contexts especially highlight digital well-being as a developmental concern. Cao and Li (2023) argue that for young children, digital well-being hinges on managing screen time and ensuring that digital play complements, rather than replaces, physical and social activity. Johnston (2021) supports this view, noting that immersive digital play must be balanced with real-world interaction to avoid negative effects on emotional development. Guidelines from Gupta et al. (2022) further emphasize age-appropriate screen limits and the importance of preserving routines related to sleep, exercise, and interpersonal relationships. Meanwhile, the social and relational dimension of digital well-being concerns how technology affects human connection. Although digital tools can enhance connectivity, they also risk displacing in-person interaction, particularly when they dominate daily routines (Smits et al., 2022).

Importantly, scholars advocate for an integrated, multidimensional approach. Smits et al. (2022) propose a fourfold model encompassing a healthy body (*physical dimension*), healthy mind (*mental*), happy self (*emotional*), and social self (*connectedness*). This framing suggests that digital well-being is not merely about reducing screen time but about fostering environments that support holistic health and human flourishing. Thomas et al. (2022) echo this, calling for proactive interventions—such as digital detox tools, public awareness campaigns, and wellness-by-design technologies—to address the growing impact of digital overload. Together, these studies converge on a comprehensive understanding of digital well-being: a dynamic, context-dependent state that is shaped by individual habits, environmental design, and broader socio-technical systems.

### 2.4. Digital Competencies, Citizenship, and Prosocial Values in Youth Education

As digital well-being becomes a central concern in educational contexts, particularly in higher education, fostering digital competencies and a strong sense of digital citizenship has emerged as essential for preparing youth to engage ethically and responsibly in online environments. Digital competencies encompass not only technical skills but also critical thinking, media literacy, and the capacity to manage one’s digital identity and well-being (Smits et al., 2022). In parallel, digital citizenship refers to the ability to participate actively and responsibly in digital life—respecting others, protecting privacy, and navigating complex social dynamics in virtual spaces (Dewitz, 2022). Cultivating these competencies is not only crucial for academic and professional success, but also for promoting prosocial behavior in digital settings, where anonymity and rapid communication can sometimes facilitate incivility, misinformation, or exclusion. Higher education institutions play an important role in embedding digital ethics, empathy, and civic engagement into curricula, helping students move from passive consumption to reflective, values-driven participation (Büchi, 2024; Bhattacharya et al., 2023). Notably, adolescents and university students are particularly vulnerable to the physical effects of digital overuse—including poor sleep hygiene, screen fatigue, and musculoskeletal strain—due to prolonged and often unregulated engagement with devices for study and socialization (Almourad et al., 2021; Thomas et al., 2022). By integrating digital well-being with digital literacy and character education, universities can create inclusive learning environments that foster resilience, collaboration, and responsibility—key ingredients for shaping informed, compassionate digital citizens. This holistic approach aligns with the broader goal of empowering young people to thrive in interconnected societies while safeguarding their own and others’ well-being.

Digital citizenship, while increasingly central in youth education, remains a multifaceted and evolving concept—particularly when connected to experiential and civic models such as service-learning. Initially defined in instrumental terms as the understanding of digital etiquette and appropriate use of technology (Ribble & Bailey, 2004), the concept has since expanded to encompass more political and critical dimensions. Choi (2016), for example, reframes digital citizenship as the ability to position oneself actively and ethically within a digital society, emphasizing not just individual skills but also collective agency, critical thinking, and civic engagement. In this view, the digital citizen is not merely a competent user but an empowered participant who employs technology to improve social contexts, advance public discourse and exercise responsibility in digital environments. As such, digital citizenship comprises three interrelated dimensions: active engagement, informed and critical thinking, and social responsibility, all rooted in prosocial values and participatory ethics. In this framework, service-learning offers a powerful pedagogical strategy for developing digital citizenship, as it promotes both curricular and transversal competences through real-world civic engagement.

## 3. (e-)Service-Learning as an Educational Approach to Digital Well-Being: Three Case Studies

### 3.1. Method

The increasing integration of digital technologies into education has brought renewed attention to the concept of digital well-being, particularly within pedagogical practices that seek to combine academic learning with civic engagement. SL and e-SL represent promising educational approaches for fostering this integration. This paper presents a qualitative analysis of three higher education case studies that explicitly address digital well-being through SL or e-SL methodologies.

To identify the case studies, we conducted a non-systematic, thematic literature review focused on the intersection of four core concepts: “*service-learning*”, “*e-service-learning*”, “*digital well-being*”, and “*higher education*”. The review process—so-called literature-based case study analysis—included targeted searches in academic databases such as Scopus, Web of Science, and ERIC, using combinations of the aforementioned keywords, without restrictions related to time frame or geographic area. Given the limited number of publications addressing all four dimensions simultaneously, we extended the search to include sector-specific repositories, such as the website of the European Observatory of Service-Learning in Higher Education (EOSLHE). This broader approach enabled us to identify three case studies from diverse institutional and geographical contexts. These cases were selected based on their explicit integration of digital well-being into SL or e-SL practices, their potential for generalizability, and their alignment with experiential and civic learning principles.

To guide the descriptive analysis, we developed an analytical matrix structured around five main categories: (1) the nature and structure of the SL or e-SL experience; (2) the use of technology as an educational tool, including its integration into the learning process and intended pedagogical purposes; (3) technology as an explicit educational content, either as a macro-level learning goal or as content related to digital well-being, digital citizenship, and critical digital literacy; (4) technology as an experiential environment that supports reflection, communication, and civic engagement; and (5) the dimension of impact and transformation at both the individual and community levels.

### 3.2. Results

The selected cases varied in format (in-person, blended, and online), emphasized real-world impact and aligned with key dimensions of digital education. Although based on secondary data, the selected cases were analyzed using a structured analytical matrix to draw interpretive insights and identify common patterns, tensions, and pedagogical implications related to digital well-being.

The descriptive analysis of the three cases is presented below (Table 1):

1. “Safe Friend is Always a Trend!”—University of Zagreb. This in-person SL initiative (Mikelić, n.d.) involved university students from Information Sciences collaborating with a local primary school, which acted as the community partner and “recipient” agency. The project addressed the school’s expressed need to promote digital safety and well-being among children. University students co-designed and delivered digital education activities for primary school pupils, focusing on online threats such as cyberbullying and identity misuse. Tools such as Prezi, Google Sites, and e-portfolios were used to co-create interactive content. In this context, technology served both as a civic engagement medium and as the core instructional content. The collaboration was intergenerational and dialogic, with teachers, children, and university students engaged in reciprocal learning. The partnership was designed to be reciprocal rather than transactional, aligning with the SL principles of mutual benefit, civic engagement, and co-construction of knowledge. Reported student outcomes included enhanced digital literacy, civic responsibility, and communication skills.
2. “Promotion of Children and Adolescent Development”—Hong Kong Polytechnic University. This SL project was transformed from a face-to-face mode to an online format during the COVID-19 pandemic (Zhu et al., 2022). It involved university students from the Economics and Management field collaborating with local schools and youth service organizations, which acted as community partners. The project was designed to foster well-being and meaning in life (MIL) among children and adolescents from socio-economically disadvantaged contexts. Most activities were conducted online, with students delivering development-focused sessions and interactive content aimed at promoting emotional resilience and personal growth. Reported outcomes included significant improvements in students’ psychological well-being, life satisfaction, and positive youth development indicators such as cognitive and behavioral competence and positive identity. The initiative also led to a revision of digital tools and strategies to ensure the inclusion and effectiveness of activities for individuals from marginalized backgrounds.
3. “Integrating Social-Emotional Learning (SEL), Digital Wellbeing and Engagement”—Turkmenistan (Nuriveya et al., 2024). This is a blended service-learning project involving 142 university students who participated in a semester-long course not directly linked to a specific academic subject but designed as a cross-cutting educational experience. The e-SL initiative was carried out in collaboration with local educational and youth development organizations, which acted as community partners. The e-SL project introduced an innovative approach to social-emotional learning (SEL) by embedding SEL competencies directly into the design and operation of hybrid digital platforms. These platforms were not only used for cognitive learning, but also designed to nurture emotional awareness, interpersonal competence, and civic engagement. Reported outcomes included improved problem-solving and communication skills, increased empathy and teamwork, and a deeper understanding of social issues. The initiative also fostered a stronger sense of civic responsibility among participating students. Researchers enriched their study by adding a particularly deep insight into two participants, Lina and Adi, through a qualitative case study that confirmed their findings and underlined that the so-called emotion-tracking tool and also the private journal were effective ways to gain confidence, form meaningful digital interactions and promote emotional awareness.

## 4. Quality Standards of e-Service-Learning and Digital Well-Being in Higher Education

Although e-SL necessarily entails pedagogical decisions tailored to specific disciplinary contexts, scholars such as Furco and Norvell (2019) and Lucas and Thomas (2021) have argued for the importance of a unifying framework that can provide cross-cutting guidance for its design and implementation. In response to this need, Culcasi et al. (2023) have developed the *e-SL Design Framework*, a conceptual and operational tool designed to help university faculty, instructional designers, and community partners create effective and context-sensitive e-SL experiences.

As a pedagogical model that evolves in dialogue with the changing landscape of higher education, the *e-SL Design Framework* offers a flexible yet robust roadmap for integrating e-SL into academic programs. It fosters a comprehensive and interconnected vision of education, one that supports the meaningful, sustainable, and holistic adoption of digital service-learning practices within contemporary universities. Indeed, technology here is envisioned not simply as an instrument but as a dynamic enabler of transformative learning.

The development of the *e-SL Design Framework* has paved the way for identifying a set of quality standards for e-SL (e-SL4EU Consortium, 2022a), an essential step in understanding how to effectively operationalize this approach and unlock its transformative potential. These standards—developed within the Erasmus+ project e-SL4EU (2021–2024)—serve not only as practical guidance for implementation but also as analytical tools for discerning the key “ingredients” that enable genuinely transformative experiences, beyond the specificities of any given disciplinary or institutional context. In the academic literature, quality standards are typically defined as a collection of measurable indicators that help assess whether the desired educational and service-related outcomes have been achieved. They function as progressive metrics, capable of capturing both the development and the impact of SL initiatives over time. Importantly, their “progressive” nature shifts the focus toward the core dimensions that characterize this pedagogical model, emphasizing depth, intentionality, and the real-world effects of educational practices.

The e-SL4EU (2021–2024) quality standards are grounded in the presented Framework, offering a methodological evolution designed to ensure educational quality even in remote or hybrid settings. These standards are organized into several key clusters (Table 2): meaningful learning; relevant service; active student engagement; systematic reflection; purposeful integration of technology; and participatory assessment (e-SL4EU Consortium, 2022b).

Compared to traditional SL standards (e.g., National Youth Leadership Council, 2008), the e-SL4EU model introduces innovative elements such as *integral education*, which promotes the development of 21st-century skills (e.g., critical thinking, creativity, and intercultural communication), deeply connected to the *technological coherence*: the ethical, motivating, and pedagogically sound use of digital technologies throughout the entire learning cycle (Culcasi et al., 2023). This underscores the notion that, even when technology in e-SL serves merely as a channel or means to reach communities, its use must be critically and meaningfully chosen to align with the underlying rationale for its implementation. Particular attention is also devoted to technology-mediated reflection, which is addressed across its personal, social, and professional dimensions, as well as to the centrality of community relationships, which must remain authentic, collaborative, and reciprocal. Furthermore, the standards emphasize the importance of a *multi-level assessment* approach that considers not only the educational and social impact of the experience but also its technical and digital components, encouraging moments of public sharing and collective celebration with the community (e-SL4EU Consortium, 2022a). Taken together, these elements contribute to shaping an innovative European standard for e-SL, one that is responsive to the evolving demands of contemporary higher education and to the broader transformations reshaping educational practice in the digital age.

In light of the literature presented in this review and the case studies analyzed, this paper presents a further development of the standards established in the e-SL4EU project. In particular, all clusters have a specific connection with aspects of digital well-being:

- Within the first cluster (*meaningful learning*), we introduced a specific focus on digital well-being that, in a holistic perspective (Escofet, 2025), should include a comprehensive understanding of digital well-being that encompasses physical, emotional, cognitive, and social dimension.
- In the second cluster (*relevant service*), we included the idea of digital empowerment that should foster opportunities to ensure equitable access to digital infrastructure and provide technical and pedagogical support to all participants in order to fully engage them and develop their potential, particularly those from marginalized backgrounds (Aramburuzabala et al., 2024).
- Within the fourth cluster (*systematic reflection*), we specify that reflection may take place through technology as well as about technology itself, emphasizing its critical and meaningful integration. Furthermore, a specific quality criterion related to digital well-being is developed.
- Within the sixth cluster (*participatory assessment*), an additional quality standard is introduced concerning the dimension of digital citizenship, as the development of competences oriented toward the common good represents the highest aim of service-learning.

## 5. Discussion

Analysing the three case studies alongside the theoretical foundations discussed in Section 2.1, Section 2.2, Section 2.3 and Section 2.4 highlights both common principles and different strategies for incorporating digital well-being into SL and e-SL practices in higher education. As explored in Section 2.3, digital well-being is a multidimensional concept encompassing mental, emotional, relational, and physical health (Smits et al., 2022; Büchi, 2024). All three projects recognize digital well-being as a vital educational and civic goal, demonstrating the potential of SL/e-SL to serve as transformative pedagogies that connect experiential learning with responsible digital engagement.

The three cases reveal shared commitments, such as using digital technologies for civic purposes, integrating reflective practices, and cultivating digital citizenship (see Section 2.4). However, they differ significantly in degree of technological integration and format:

- **Technology as curricular content of the SL/e-SL project:** When considering technology as educational content—that is, as a topic explicitly addressed within the learning process—this dimension is most evident in the Zagreb and Turkmenistan projects, where issues such as digital well-being, cyberbullying, and safe browsing were central themes. The Hong Kong initiative, while primarily shaped by its online learning context during the pandemic, also incorporated elements of technology as content by promoting healthy digital practices and fostering psychological and emotional well-being. In addition, in the Turkmenistan case, technology was not only content but also an explicit project objective, as students co-designed hybrid platforms integrating social-emotional learning with digital engagement tools.
- **Technology as a tool or product of the SL/e-SL project:** In the “Safe Friend is Always a Trend!” project (University of Zagreb), technology was employed both as an educational tool and as the core content of the SL experience. The use of digital platforms was intentionally tied to both the learning goals and the service objectives of the project. Therefore, this case clearly aligns with the category of *integrated channel* (Culcasi et al., 2022b), as technology was a vehicle for civic education and co-production of knowledge, and with elements of the *instrumental objective* model, given that the final product—interactive digital content—was itself a resource with civic value. The “Promotion of Children and Adolescent Development” project (Hong Kong Polytechnic University) began as a traditional face-to-face SL experience and was later adapted to a fully online format in response to the COVID-19 pandemic. In this context, technology served as more than a logistical solution: university students used digital platforms to design and facilitate structured sessions aimed at promoting emotional resilience, well-being, and meaning in life among socio-economically disadvantaged children and adolescents. While the core content was not explicitly digital in nature, the intentional use of technology to support inclusion and engagement makes this case a strong example of the *integrated channel* category (Culcasi et al., 2022b). The initiative also led to the revision of digital tools and strategies to enhance accessibility and effectiveness, further supporting its alignment with this more pedagogically embedded model of technology use. The “Integrating SEL, Digital Wellbeing and Engagement” project (Turkmenistan) demonstrates a more complex and innovative integration of technology. Here, digital platforms were not only the medium, but also the pedagogical and civic space in which emotional awareness, interpersonal competence, and social responsibility were cultivated. The technology itself was co-designed and structured to facilitate social-emotional learning, making it both a learning environment and a deliberate curricular tool. This positions the case clearly within the *integrated objective* category (Culcasi et al., 2022b), as the technological component is central to the learning architecture and outcomes of the project. Rather than using pre-existing tools for content delivery, the platforms themselves were designed to embody and foster the intended SEL and civic competencies.
- **Technology as a learning environment of the SL/e-SL project:** “Safe Friend is Always a Trend!” from University of Zagreb is in-person; “Promotion of Children and Adolescent Development” from Hong Kong Polytechnic University was transformed from face-to-face to fully online during the COVID-19 pandemic; and the project from Turkmenistan integrates social-emotional learning (SEL) into a blended SL model.

The three case studies differ not only in technological integration and project content, but also in their pedagogical format. These variations invite a broader reflection on how technology interacts with SL practices. Rather than considering electronic service-learning (e-SL) as a separate category within the broader SL landscape, we suggest that it may be more accurately understood as the central point of a pedagogical continuum. At one end lies traditional, fully on-site SL, and at the other, fully online or “extreme e-SL” (L. S. Waldner et al., 2012). Analyzing diverse case studies along this continuum challenges the binary distinction between SL and e-SL and encourages a redefinition of the concept itself. From a technological standpoint, e-SL is not simply the online version of SL, but a broader pedagogical paradigm encompassing any SL experience where technology plays a meaningful role— as a learning environment, as curricular content, or as a civic tool/objective. This perspective enables us to recognize that even an entirely in-person project, such as “Safe Friend is Always a Trend!”, can be framed within an e-SL logic if technology is central to the project’s educational content and civic mission. Similarly, the Hong Kong project, though shaped by pandemic-related necessity, used digital platforms in an intentional and pedagogically embedded way, making it more than just an adaptation of SL to an online format. In the Turkmenistan case, the technological infrastructure itself became both the object and medium of learning, exemplifying the integrated level of digital engagement within SL practice. In this view, the term e-SL becomes less about format and more about intentionality. It reflects the depth and purpose with which technology is integrated into educational, relational, and civic dimensions of learning. As such, the distinction between SL and e-SL may no longer be necessary, since many in-person SL initiatives are increasingly shaped by digital tools, platforms, and issues. Embracing this continuum-based understanding offers a more nuanced framework for analyzing and designing SL practices in the digital age.

These variations reflect different institutional priorities and pedagogical contexts, but converge in illustrating how SL/e-SL can embed digital well-being into real-world, reflective learning processes. Importantly, the integration of artificial intelligence (AI) remains marginal in these cases. However, as outlined in Section 2.2 and in recent literature (Veldhuis et al., 2025), AI has the potential to reshape both the implementation and the ethical dimensions of SL projects. From adaptive feedback systems to AI-supported community mapping or early diagnostics (as in the IARA project), future SL/e-SL models could explore how AI technologies contribute to or challenge digital well-being goals.

Three overarching insights emerge from the cross-case analysis:

- **Technology as content, tool, and context**—As Section 2.2 highlights, digital technologies (including AI) should be viewed not only as instruments but as integral elements of the learning process. This includes fostering ethical reflection and civic responsibility in their use.
- **Reflective and relational learning**—Building on the foundations of SL and e-SL (Section 2.1 and Section 2.4), all three cases promoted structured reflection on students’ digital behaviors, emotional responses, and civic values, enabling both personal and social transformation.
- **Diversity of implementation**—The examined cases demonstrate that digital well-being can be effectively pursued across various pedagogical formats (in-person, blended, and online), reflecting the adaptability and resilience of the SL/e-SL model.

These findings also resonate with the entangled pedagogy framework (Fawns, 2022), which critiques binary distinctions between pedagogy and technology. Instead, it promotes an integrated vision, where technologies—including AI—are embedded in ethical, context-aware, and learner-centered educational experiences.

In summary, the case studies confirm that promoting digital well-being through SL/e-SL requires not only technical infrastructure as well as intentional pedagogical strategies, aligned with the quality standards discussed in Section 4. These include holistic student development, equitable digital access, ethical technology use, and meaningful community collaboration. As digital—and increasingly AI-mediated—environments shape higher education, the design of SL/e-SL projects must evolve accordingly to ensure that civic engagement and well-being remain at the heart of digital learning experiences.

The quality standards presented in this paper build upon the existing e-SL4EU Design Framework, which was originally developed in the immediate aftermath of the COVID-19 pandemic, when the role of technology in SL was still underexplored. In recent years, research on the intersection between SL and digital environments has grown significantly, although much of this work remains predominantly theoretical. Our review revealed a noticeable lack of empirical studies and detailed case-based documentation illustrating how digital technologies and digital well-being are concretely integrated into SL or e-SL practices. This scarcity highlights an important gap in the academic discourse.

Despite their limited number, the three selected case studies offer valuable insights into the embedding of technology in SL/e-SL initiatives. They highlight how technology can serve as a learning environment, as curricular content, and as an explicit objective of the educational process. As such, they provide a useful lens for critically reflecting on and updating key components of the e-SL4EU Framework. Moreover, the findings underscore the importance of maintaining a practice-oriented and context-sensitive approach to SL/e-SL, one that is responsive to evolving social challenges such as digital inclusion, citizenship, and well-being.

In this perspective, the role of technology in SL/e-SL must be continuously re-examined—not only in terms of tools and platforms, but also in light of the social meanings, ethical questions, and pedagogical purposes it entails. These considerations align with the SL imperative to respond to emerging needs in real-world contexts through educational strategies that are both reflective and transformative.

## 6. Conclusions

In recent years, the rapid evolution of digital technologies and social media platforms has transformed the way individuals interact and engage with their communities. In this context, SL has emerged as a powerful pedagogical approach to promoting digital well-being. SL projects can be intentionally designed to involve students in responsible, inclusive, and reflective digital practices, serving as effective strategies for both intervention and prevention. Despite growing academic interest in both SL and digital well-being, their intersection remains underexplored. As outlined in Section 2.1, Section 2.2, Section 2.3 and Section 2.4, much of the existing literature focuses on either the pedagogical value of SL or the challenges of digital engagement, without systematically investigating how SL—especially in online or hybrid modalities—can serve as a vehicle for fostering responsible, inclusive digital behavior. This study addresses this gap by analyzing three international case studies that operationalize digital well-being within SL and e-SL projects. Based on this analysis and informed by the e-SL4EU Framework (Section 4), we propose an updated conceptual model that positions digital well-being as a core dimension of quality in SL design.

This dual contribution—empirical and theoretical—reinforces the idea that technology should not only be a vehicle for educational delivery, but also a topic for critical inquiry and a context for cultivating digital citizenship. While the role of artificial intelligence was not a central theme in this study, its educational potential and ethical implications should be a priority in future research. AI, if used responsibly, may enhance digital well-being by supporting personalization, feedback, and data-informed reflection—but it may also exacerbate inequalities, privacy concerns, and psychological stress if uncritically integrated.

The findings show that SL/e-SL can be a powerful strategy for nurturing a responsible digital culture. By embedding digital well-being, reflective practices, and social-emotional learning within academic programs, universities can equip students not only to engage meaningfully with their communities but also to thrive in increasingly complex digital ecosystems. This aligns with the holistic educational goals proposed by United Nations Educational, Scientific and Cultural Organization [UNESCO] (2021) and with recent calls for pedagogical models that foster inclusion, resilience, and digital empowerment (Aramburuzabala et al., 2024).

Practically, this means that higher education institutions must invest in cross-disciplinary collaboration, updated teacher training, and equitable access to digital infrastructure. In particular, teacher preparation must address persistent digital divides and promote confident, critical, and ethical engagement with new technologies—AI included (Velander et al., 2024; Veldhuis et al., 2025). Without such efforts, educational innovations risk reinforcing exclusion, rather than fostering inclusion and well-being.

Future research should examine how SL/e-SL programs that include AI components (e.g., automated feedback, learning analytics, or AI-supported community services) influence students’ digital well-being, sense of agency, and ethical awareness. In parallel, longitudinal studies could investigate whether such programs contribute to reducing digital stress and enhancing mental health and civic engagement over time.

In conclusion, this review demonstrates the transformative potential of SL/e-SL to address both societal and technological challenges. By aligning SL/e-SL with digital well-being and, in the near future, with AI literacy and ethics, educators and institutions can create inclusive, participatory, and forward-looking learning environments. These environments empower students to act not only as competent digital users, but also as ethical citizens capable of shaping a just and digitally sustainable future.

## Figures and Tables

**Table 1:** Descriptive Analysis of three digital well-being SL/e-SL case studies.

Categories of Analysis and Indicators	Case 1 Croatia	Case 2 China	Case 3 Turkmenistan
**SL or e-SL** *indicators: types of experience and* *students involved*	*Type of experience:* Fully in-person SL.	*Type of experience:* e-SL during the COVID-19 pandemic.	*Type of experience:* Blended (e-)SL.
	*Participants:* Students from Information Sciences at the Faculty of Humanities and Social Sciences, University of Zagreb.	*Participants:* Students from the Economics and Management fields at Hong Kong Polytechnic University.	*Participants:* Students from various academic disciplines participated in an e-SL course at the University of Turkmenistan.
**Technology as an** **education tool** *indicators: digital tools used* *and purposes*	*Tools*: Google Sites, Prezi, e-portfolios, and reflective journal.	*Tools*: online platforms, forums, and digital presentations.	*Tools*: social media, digital platforms, and digital advocacy tools.
	*Purposes*: dissemination, reflection, awareness, and engagement.	*Purposes*: online workshops, video teaching, virtual tours, group reflection, and engagement.	*Purposes*: social-emotional learning (SEL) modules, online discussions, storytelling, and social media campaigns.
**Technology as a** **curricular content** *indicators: objectives* *and contents*	*Objectives*: explicit objectives related to digital well-being. Focus on youth cyberbullying and social media misuse.	*Objectives*: explicit objectives related to well-being and meaning in life (MIL). Focus on the promotion of the subject.	*Objectives*: digital domain as growth and challenge and design of digital platforms.
	*Contents*: internet safety, cyberbullying, privacy, and responsible social media use.	*Contents*: student self-awareness, healthy tech habits, and prosocial behaviors.	*Contents*: social-emotional learning, digital well-being and engagement, safe browsing.
**Technology as an** **experiential context** *indicators: relationship* *and reflection*	*Relationships*: relationships among students and project’s partners were developed on site during 45 h of field work.	*Relationships*: relationships with community partners were developed in non-face-to-face mode.	*Relationships*: hybrid platforms offered a unique opportunity to extend SEL and relationships beyond the physical classroom into a blended environment.
	*Reflection*: students reflected on the SL experience using digital tools (e.g., e-portfolio).	*Reflection*: students reflected on the e-SL experience using digital tools.	*Reflection*: students’ reflection and emotional well-being were supported in hybrid settings.
**Impact and** **transformation** *indicators: community outcomes* *and students outcomes*	*Community outcomes*: primary pupils and their parents learned cyberbullying and internet security with an emphasis on social networks and responsible behavior on the internet.	*Community outcomes*: revision of tools and strategies to ensure inclusion and effectiveness for individuals from socio-economically disadvantaged contexts.	*Community outcomes*: new digital platforms not only for cognitive learning but also for nurturing emotional awareness and interpersonal competencies.
	*Students’ outcomes*: university students gained IT skills, presentation skills, communication, and civic skills. They engaged in active citizenship.	*Students’ outcomes*: significant improvements in psychological and subjective well-being, as well as in their sense of meaning in life.	*Students’ outcomes*: problem-solving skills, empathy, communication, teamwork, and a deeper understanding of social issues.

**Table 2:** Quality standards for e-service-learning practice.

Cluster	Quality Standards	Description
**1.** **Meaningful learning and digital well-being**	*Relevant learning*	It provides learning opportunities within a community context and fosters an understanding of digital well-being complexity.
	*Curricular integration* *Integral education*	It defines digital learning objectives, skills, and values in alignment with the academic curriculum. It promotes the development of 21st-century competencies (soft skills) such as digital critical thinking and digital communication.
	*Strategies for achieving learning goals*	It employs active, evidence-based digital teaching strategies appropriate to the nature and goals of the project, clearly defining students’ roles and responsibilities in achieving learning outcomes.
	*Holistic conception of digital well-being*	It adopts a comprehensive understanding of digital well-being that includes physical, emotional, cognitive, and social dimensions.
**2.** **Relevant service**	*Social need*	It addresses real digital needs through clear and measurable objectives, jointly defined by students and the community.
	*Meaningful interaction*	It fosters meaningful interactions—even remotely—with physical, social, or virtual communities.
	*Reciprocity*	It is grounded in authentic and equitable partnerships between academic institutions and communities.
	*Digital empowerment*	It offers ongoing guidance to enable all participants to fully engage, particularly those from marginalized backgrounds.
**3.** **Active student engagement**	*Student voice*	It recognizes and promotes students’ voices and their active digital engagement as integral members of the community.
	*Ownership and responsibility*	It promotes students’ sense of digital responsibility by engaging them in demanding tasks and involving them throughout all stages of the project.
	*Adequate duration and intensity*	It provides sufficient time, in terms of both duration and intensity, to enable meaningful digital learning experiences.
**4.** **Systematic reflection**	*Time for reflection*	It encourages structured reflection throughout all stages of the project—before, during, and after—to help articulate and make explicit tacit knowledge in the digital area.
	*Dimensions of reflection*	Reflection encompasses the personal, social, and curricular/professional dimensions of the experience. It may take place through technology as well as about technology itself, emphasizing its critical and meaningful integration.
	*Relational and social awareness about digital technologies use*	It includes reflective components that help students and community members assess how digital technologies shape their relationships, communication patterns, and co-creation of knowledge, encouraging practices that prioritize authentic human connection.
**5.** **Purposeful integration of technology**	*Ethical and creative use of technology*	It promotes the use of technology in an ethical, creative, and pedagogically meaningful way to effectively support learning processes.
	*Coherent technological integration*	It ensures a structured and seamless integration of digital and face-to-face components, maintaining consistency across different modes of delivery.
	*Goal-oriented technological tools*	It employs digital tools that are appropriate to the nature and objectives of the planned activities, avoiding one-size-fits-all solutions.
	*Ongoing technical and pedagogical support*	It provides continuous support—both technical and pedagogical—to facilitate actors’ engagement with the digital environment.
**6.** **Participatory assessment**	*Project evaluation*	The project is assessed by students, community partners, and academic institutions using multi-perspective indicators.
	*Celebration and dissemination*	The outcomes are documented and publicly shared through collaborative events that involve all stakeholders.
	*Assessment of integral development*	The project evaluates students’ personal and professional development—including digital awareness—as part of their overall learning experience.
	*Technical components*	The project assesses the effectiveness and impact of its technological and digital elements.
	*Digital citizenship*	It helps learners navigate digital spaces critically, manage their digital presence responsibly and recognize the broader societal impact of their online behavior.

## Data Availability

Not applicable.
